# The Association of Levels of and Decline in Grip Strength in Old Age with Trajectories of Life Course Occupational Position

**DOI:** 10.1371/journal.pone.0155954

**Published:** 2016-05-27

**Authors:** Hannes Kröger, Johan Fritzell, Rasmus Hoffmann

**Affiliations:** 1 European University Institute, Florence, Italy; 2 Aging Research Center, Karolinska Institutet & Stockholm University, Stockholm, Sweden; Indiana University Richard M. Fairbanks School of Public Health, UNITED STATES

## Abstract

**Background:**

The study of the influence of life course ***occupational*** position (OP) on health in old age demands analysis of time patterns in both OP and health. We study associations between life course time patterns of OP and decline in grip strength in old age.

**Methods:**

We analyze 5 waves from the Survey of Health Ageing and Retirement in Europe (n = 5108, ages 65–90). We use a pattern-mixture latent growth model to predict the level and decline in grip strength in old age by trajectory of life course OP. We extend and generalize the structured regression approach to establish the explanatory power of different life course models for both the level and decline of grip strength.

**Results:**

Grip strength declined linearly by 0.70 kg (95% CI -0.74;-0.66) for men and 0.42 kg (95% CI -0.45;-0.39) for women per year. The level of men’s grip strength can best be explained by a critical period during midlife, with those exposed to low OP during this period having 1.67 kg (95% CI -2.33;-1.00) less grip strength. These differences remain constant over age. For women, no association between OP and levels of or decline in grip strength was found.

**Conclusions:**

Men’s OP in midlife seems to be a critical period for the level of grip strength in old age. Inequalities remain constant over age. The integration of the structured regression approach and latent growth modelling offers new possibilities for life course epidemiology.

## Introduction

Life course research in social epidemiology has often considered either time-patterns of socio-economic position (SEP) as predictors for static measurements of physical function, or static measurements of SEP as determinants of levels and change in physical function in old age. In the first type of study, the objective is often to identify the influence of different trajectories of SEP on health in later life. Many studies try to distinguish between models of accumulation, critical periods, or social mobility. While some authors have argued that this distinction might never be possible[1], other approaches have been developed that treat discriminating between models as an empirical problem[2–6]. The accumulation model proposes that in each period in the life course risk factors can influence health, and that resulting health inequalities accumulate over time. The critical or sensitive period models stress the importance of a particular time window in the life course (often childhood) as the main or even sole period in which SEP exacts an influence on health later in life[7]. Whereas the stronger version of this model assumes irreversibility, the softer version, often referred to as sensitive period model, note that later events can modify the effects of the earlier exposure. Alternatively, social mobility models stress possible health effects of both inter- and intra-generational upwards or downwards social mobility. These studies take a dynamic perspective on SEP or other risk factors, but predominantly a static perspective on the health outcome [8–12]. The second type of studies often use indicators from a certain point in time, or time-constant indicators of SEP, to predict trajectories of health[13–15] Moreover, few of these studies focus on health at old age. If the developments are investigated with respect to health inequalities, three general scenarios can be expected. The *age-as-leveler* hypothesis predicts decreasing inequalities with higher age due to selective mortality, lack of further exposure to poor working conditions, and the overriding influence of biological aging[16,17]. Proponents of *cumulative (dis)advantage* would expect existing inequalities to increase as certain factors like health behavior or living conditions continue to work as drivers of health inequality in old age[18]. Lastly, it is possible that health inequalities remain largely stable throughout old age.

We endeavor to combine these two perspectives to investigate the association between trajectories in occupational position (OP) throughout the life course and levels and decline of grip strength. This combined approach yields a more comprehensive picture of the interplay between trajectories of OP and physical function in old age[19]. We estimate the association between levels of and decline in grip strength with life course OP for all possible trajectories separately, and test patterns in these trajectories according to the established models of accumulation, critical periods, or social mobility. For this purpose, we generalize the structured regression approach[20] to the framework of structural equation modeling (SEM) to be compatible with the prediction of differences in intercept and slope of a latent growth model (LGM).

Grip strength has become a popular indicator of physical functioning in surveys. It is both indicative of overall muscle and physical functioning [21]. Physical functioning in old age is an important prerequisite for independence, quality of life and for avoiding comorbidities due to inhibited mobility [22]. Grip strength is objectively measured, avoiding biases that might arise in self-reports. It is further predictive of mortality, showing that it is related to health status more generally. Further, its measurement has no relevant floor or ceiling effects. This means that improvement or worsening of the indicator is possible at almost all levels of the measurement. This is especially important if individual decline is to be measured and a great advantage over scales of physical functioning like activities of daily living (ADL) in surveys which do have floor and ceiling effects.

Our study contributes to life course epidemiology: firstly, by integrating theories on life course models of exposure and change in health inequalities; secondly, by providing empirical results from a large European dataset; and thirdly, by illustrating a novel combination of the structured regression approach with LGM.

## Materials and Methods

The analysis is based on anonymized secondary data. The SHARE survey is subject to ethical approval of Ethics Council of the Max-Planck-Society for the Advancement of Science.

The Survey of Health Ageing and Retirement in Europe consists of data on health and socioeconomic variables of non-institutionalized individuals aged 50 and older across 20 European countries[23]. We use waves 1–5, collected bi-annually between 2004 and 2013. In the third wave, retrospective life course data was collected on OP from childhood to old age[24].

The total sample of the SHARE respondents was restricted in the following way. Firstly, only participants of wave three who answered the life history questionnaire were retained. Secondly, all those who reported never having been employed, or who had missing information on childhood or adulthood OP, were excluded. The third restriction was that all individuals had to be between 65 and 90 (birth cohorts 1922 to 1938) and no longer working during the period of observation. The resulting sample consists of 3067 men and 2041 women in 13 countries taking part in wave three (Austria, Germany, Sweden, Netherlands, Spain, Italy, France, Denmark, Greece, Switzerland, Belgium, Czech Republic, Poland).

### Measures

We dichotomized OP, separating the group of *elementary occupation* (ISCO-88 major group 9) from all others. Workers in this group perform mostly simple and routine tasks, in some cases with considerable physical effort[25]. We defined three life course periods: Childhood (0–15), young adulthood (16–35), and midlife (36–64). The cut-off between young adulthood and midlife was chosen at 35, because grip strength peaks around the mid-thirties[26,27]. The indicator of exposure to low OP for childhood is the OP of the main breadwinner at age ten. For young adulthood and midlife periods the indicator refers to their own occupational status. We coded as ‘exposed to low OP’ all individuals who had worked in an elementary occupation for at least half of the years in which they reported an occupational status.

The highest grip strength measurement of two measurements per hand (dynamometer type: Smedley, S Dynamometer, TTM, Tokyo, 100kg)[28] is used as an indicator of grip strength, measured in all five waves. Grip strength has been shown to be a predictor of disability[22,29–31], morbidity[21,29,32], and mortality [33–36], and a correlate of other aspects of physical aging like frailty[37]. Furthermore, different dimensions of SEP predict grip strength[38–40], making it a useful indicator for grip strength in old age.

Grip strength is not observed for all individuals at each wave. We divided the sample into five missing value patterns (Table 1) and applied a pattern-mixture (PM) model [41] to correct for possible bias in the estimation of the LGM due to health related drop-outs or item-non-response[42]. This includes a correction for those who drop out due to death and those who are no longer able to perform the grip strength measurement. The means of the intercept and slope, and their association with life course occupational status, are estimated separately for these five groups. We also reran the analyzes using the more restrictive missing at random (MAR) assumption. The conclusions in our paper are not affected by the decision to adopt a PM over a MAR approach. We report the PM results in the main paper, because they rely on less strict assumptions than the MAR approach. We document the core results of MAR in Tables C, P and Q in S1 Appendix.

**Table 1:** Missing value patterns and participation in waves in the sample–Frequency (%).

Missing value Pattern	Men	Women
(1) Complete cases	832 (27.13)	545 (26.70)
(2) Intermittent missings	479 (15.62)	362 (17.74)
(3) No information after wave2(including drop-outs in wave 4)	891 (29.05)	503 (24.64)
(4) No information in wave 5	527 (17.18)	362 (17.74)
(5) Start at wave2, afterwards complete cases	338 (11.02)	269 (13.18)
Participation in wave 1	2112 (68.86)	1299 (63.65)
Participation in wave 2	2836 (92.47)	1895 (92.85)
Participation in wave 3	3067 (100)	2041 (100)
Participation in wave 4	2239 (73.00)	1575 (77.17)
Participation in wave 5	1784 (58.17)	1297 (63.55)
Total	3067	2041

*Note*: The estimation of the mean of the intercept, slope, and their association with life course OP is estimated separately for the 5 patterns. The weighted average is then calculated (see “Formulas for the estimation of the LGM and the generalization of SRA” in the supporting information for details).

In our model we let the initial level of grip strength (intercept) and the decline in grip strength (slope) be correlated. Positive values of the correlation indicate that those with higher initial levels of grip strength have a stronger decline, while negative values mean that initially high levels of grip strength are associated with slower decline. This means that all parameter estimates and comparisons of models in our analyses take this potential association into account and can be interpreted as after accounting for possible correlation of initial level and subsequent decline in grip strength.

All data preparation and summary statistics were conducted using Stata 14.1 with user written extensions [43,44]. The whole code necessary to replicate the analyses is available in S1 Code.

### Statistical analysis

We proceed in three steps in our analysis. First, we estimate the overall level of and decline in grip strength using LGM. We use month-specific age at each wave as an individually varying time-point[45]. The LGM is defined in the following way:

$$\begin{array}{l} y_{i}=\;\Lambda \eta_{i}+\epsilon_{i}\\ \eta_{i}=\mu_{\eta }+\zeta_{i} \end{array}$$ (1)

The index *i* refers to the observed individuals. ***y*** is the vector of the five observed measures of grip strength, ***Λ*** is the vector of constraints, identifying intercept and slope**. *ϵ***_*i*_ represents the vector of individual specific errors in grip strength. ***η***_*i*_ contains the values of each individual on the intercept and slope parameter, ***μ***_*η*_ holds the means of the intercept and slope, representing level and decline in physical functioning, and ***ζ***_***i***_ is the individual deviation from the mean of the intercept and slope, representing the variability in level and decline of physical functioning.

Second, we estimate the association between life course OP trajectories and both level and decline of grip strength. In our model we include dummy variables for each of the countries and dummies for three year cohorts. The coefficients of these dummies reflect differences in initial level and decline of grip strength between cohorts and countries. Therefore, our estimates can be interpreted as differences in level and decline of grip strength between different trajectories of occupational position, after adjusting for differences between countries and cohorts.

Third, we use the structured regression approach (SRA), which was developed to distinguish between patterns of life course exposure according to the theories of accumulation, critical and sensitive periods, and social mobility[20]. We generalize the original approach so that it can be applied in a SEM framework for the prediction of level and decline of grip strength.

The intercept (level) and slope (decline) of grip strength are predicted by exposure in the three periods of life course and all their interactions (***X***_***i***_) and controlled for differences between countries and cohorts (***C***_*i*_):

$$\eta_{i}=\mu_{\eta }+X_{i}\beta_{\eta }+C_{i}\gamma_{\eta }+\zeta_{i}$$ (2)

In the SRA, a saturated model is defined as consisting of the freely estimated effects of all periods of exposure (and all their possible interactions) on grip strength. This estimates different levels of the outcome variable for every possible trajectory of OP, yielding maximum explanatory power. If a significant association with trajectories of OP can be found in the saturated model, a set of restrictions corresponding to life course models is applied to the saturated model, and the relative model fit is assessed. For testing constraints on the coefficients predicting both levels of and decline in grip strength, we use Wald tests, which can be easily implemented into SEM, instead of the original F-test. Following a further development of the SRA, we compare the Akaike information criterion (AIC) of those models that show a p-value of over 0.1[46]. (For more technical details of the models, see S1 Appendix).

## Results

**Fig 1:**
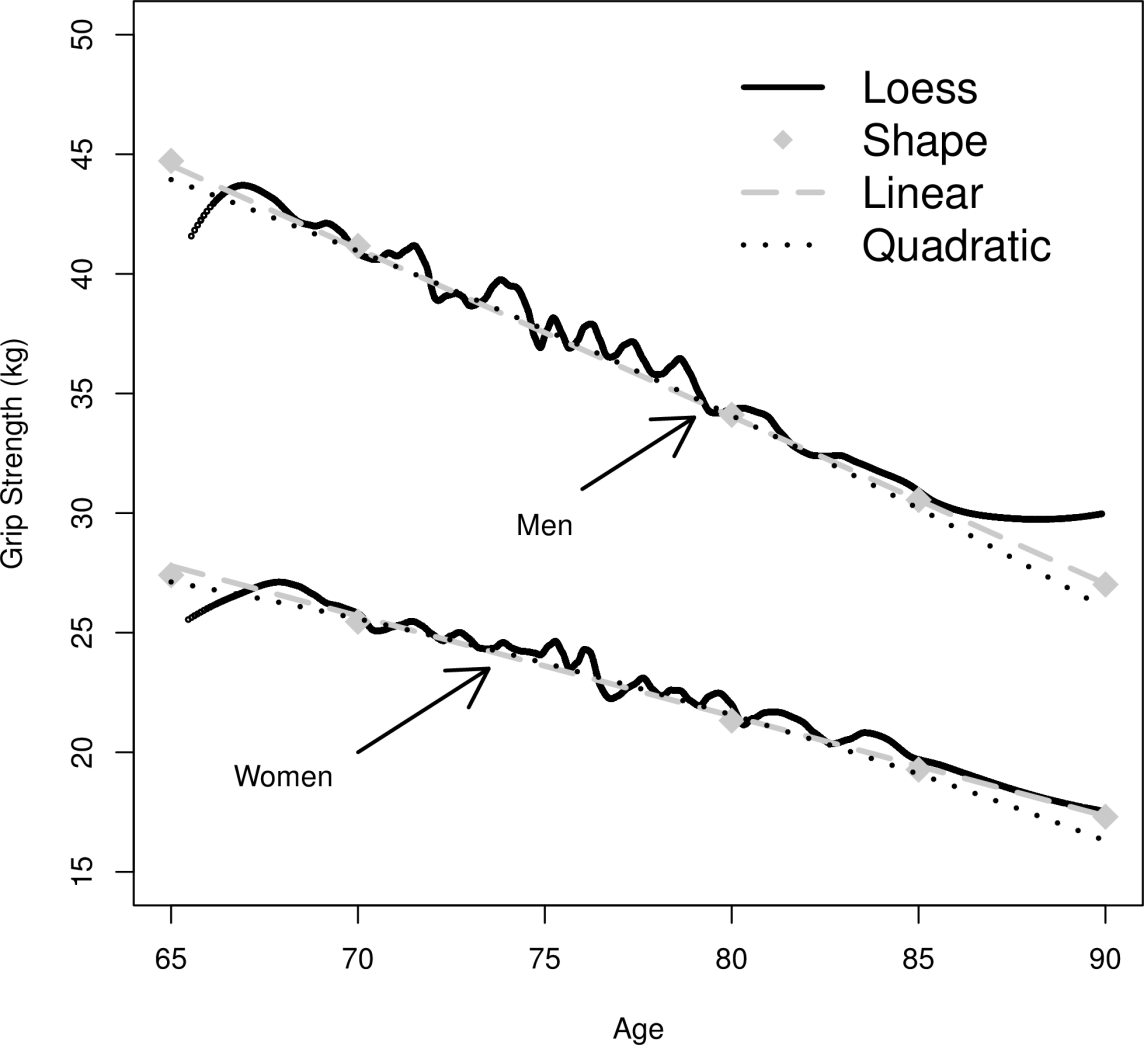
Model fit of three specifications of the slope in latent growth model of grip strength for men and women. *Note*: Figure shows the predictions of three specifications compared to the observed trajectory of grip strength estimated by locally weighted scatterplot smoothing (LOESS).

Table 2 reports the sample statistics for men and women. In the first step, we estimated LGMs for men and women to determine the shape of decline of grip strength[45]. We compared the model fit of three specifications of the growth trajectory (linear, quadratic, linear-semi-parametric). Fig 1 shows the fit of predictions of the three model specifications against the actually observed decline (locally weighted scatterplot smoothing), and Table 3 shows the BIC for the respective models. A linear decline models the trajectory of grip strength in our data set well. For men, we can observe an average grip strength of 37.54 kg (95% CI 37.28;37.80) at age 75, and an average estimated decline of 0.70 kg (95% CI -0.74;-0.66) between the ages of 65 and 90.

**Table 2:** Sample Statistics.

	Men		Women	
	Mean	Number of missing values	Mean	Number of missing values
Age at wave 1 (years)	71.77	0	71.83	0
Grip strength wave 1 (kg)	39.53	1025	24.63	805
Grip strength wave 2 (kg)	37.85	312	24.28	219
Grip strength wave 3 (kg)	36.83	244	23.09	206
Grip strength wave 4 (kg)	36.22	1156	22.65	684
Grip strength wave 5 (kg)	35.13	1615	22.03	989
Low OP in childhood (%)	19.2	0	17.05	0
Low OP in young adulthood (%)	17.87	0	22.34	0
Low OP in midlife (%)	15.91	0	20.87	0
Austria (%)	2.67	0	3.58	0
Germany (%)	7.56	0	6.96	0
Sweden (%)	8.31	0	12.1	0
Netherlands (%)	7.92	0	5	0
Spain (%)	8.74	0	4.16	0
Italy (%)	11.18	0	5.59	0
France (%)	8.38	0	11.02	0
Denmark (%)	6.94	0	11.71	0
Greece (%)	10.76	0	6.37	0
Switzerland (%)	4.73	0	5	0
Belgium (%)	11.93	0	9.75	0
Czech Republic (%)	5.48	0	11.42	0
Poland (%)	5.38	0	7.35	0
Cohorts 1922–1927 (%)	16.79	0	17.3	0
Cohorts 1928–1930 (%)	16.56	0	17.15	0
Cohorts 1931–1933 (%)	21.52	0	21.66	0
Cohorts 1934–1936 (%)	26.7	0	25.18	0
Cohorts 1937–1938 (%)	18.42	0	18.72	0
Observations	3067		2041	

**Table 3:** Model fit (BIC) of three specifications of the slope for the latent growth model of grip strength.

Slope	Linear	Quadratic	Linear semi-parametric
Men	**82256**	82276	82276
Women	**50533**	50557	50540

*Note*: Bold numbers indicate best model fit.

At the age of 75, women have an average grip strength of 23.60 kg (95% CI 23.38;23.82). During the period of observation, the annual decrease is estimated to be 0.42 kg (95% CI -0.45;-0.39). The correlation between intercept and decline in grip strength is weak for both men (0.04; 95% CI -0.09;0.17) and women (-0.11; 95% CI -0.28; 0.07), and not statistically significant (for more detailed results see Table B in S1 Appendix). This means that the initial level of grip strength is not associated with rate of decline for men or women.

**Fig 2:**
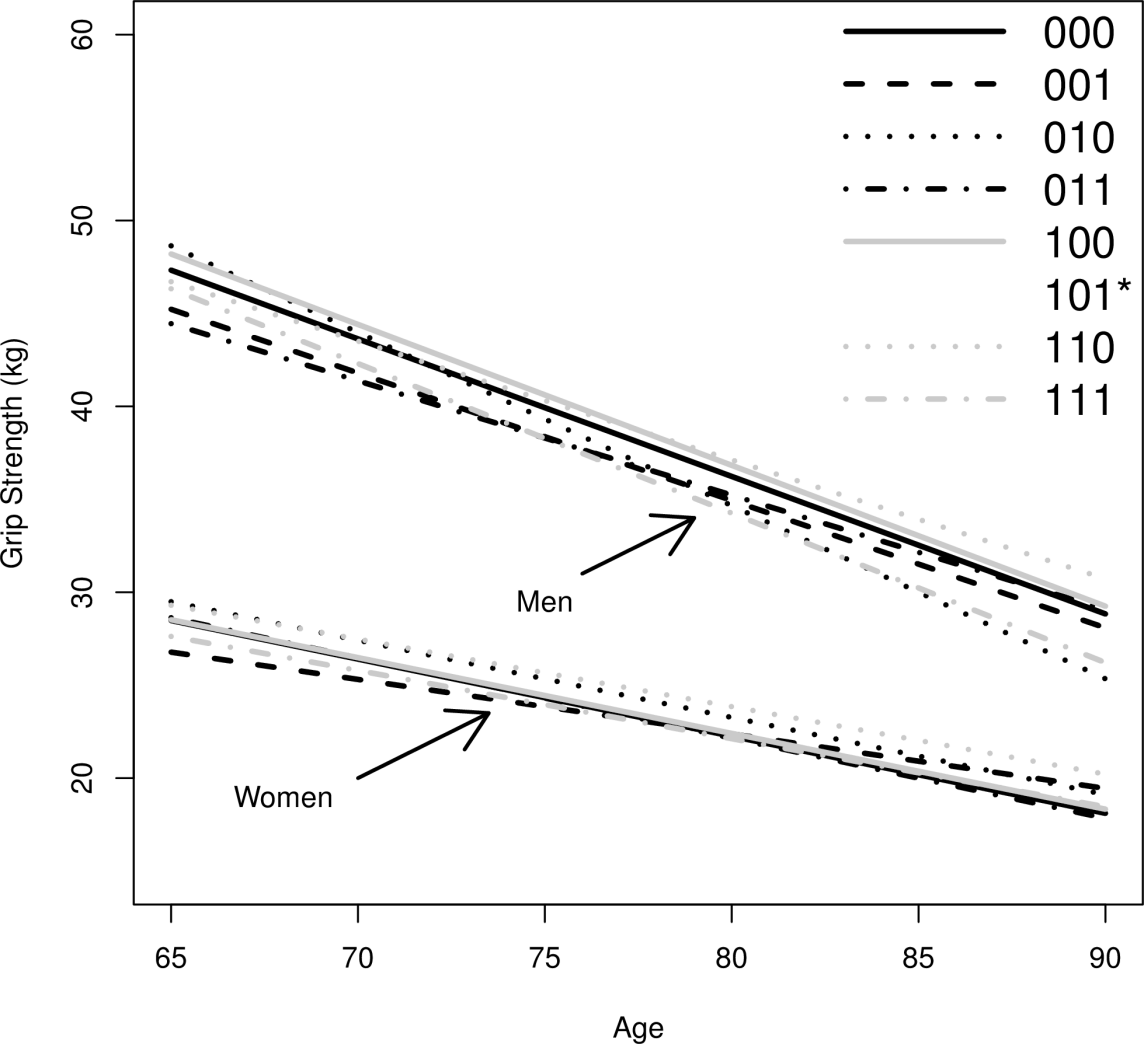
Model implied predictions of developments of grip strength by OP trajectory for men and women. *Note*: Trajectory (101) was not plotted, because the number of observations was too small to yield reliable predictions. Trajectories are described by 1 for exposure to low OP, and 0 for no exposure. The first digit indicates the status for childhood, the second for early adulthood and the third digit represents midlife. For example, 000 means always is high OP, 111 always in low OP, 001 represents downward social mobility in adulthood.

In the second step, we use life course trajectories of OP to predict the average level and the annual decline of grip strength. Fig 2 shows these predictions. The figure uses a 1 for exposure to low OP and a 0 for no exposure. Tables 4 and 5 report the respective predicted intercept and slope parameters for each of the trajectories of OP. Trajectory (101) is not plotted due to the low number of cases that make the prediction unreliable. For men, we can see that the lines run mostly parallel, although there are slight differences between the levels. A slight exception is trajectory (010), those who are exposed to low OP only in young adulthood. They are predicted to have a higher decline in physical health. From Table 4 we can see that men who achieve intergenerational upward mobility (100) have, on average, the highest grip strength, followed by those with intra-generational mobility (110) and those who are never exposed (000). The lowest levels of grip strength are found in those of opposite ***occupational*** trajectory: the inter-generationally downwardly mobile (011), the continuously exposed (111), and those who are intra-generationally downwardly mobile (001). The difference to the three highest groups is about 2 kg, which is substantial as it translates into a difference of 3 years’ decline in physical function.

**Table 4:** Predictions of intercept and slope of grip strength by life course OP pattern (kg)–Men.

Trajectory	N	(%)	Intercept (at age 75)	Slope (decline per year)
000	2125	69.29	38.26 [37.30;39.23]	-0.71 [-0.86;-0.56]
001	62	2.02	36.67 [34.53;38.81]	-0.66 [-0.92;-0.40]
010	83	2.71	37.66 [36.05;39.27]	-0.92 [-1.15;-0.69]
011	208	6.78	36.69 [35.38;38.00]	-0.60 [-0.80;-0.40]
100	315	10.27	38.93 [37.72;40.14]	-0.74 [-0.93;-0.55]
101	17	0.55	37.71 [33.73;41.68]	-0.40 [-0.87;0.06]
110	56	1.83	38.60 [36.26;40.94]	-0.60 [-0.95;-0.26]
111	201	6.55	36.60 [35.19;38.01]	-0.79 [-1.00;-0.58]
Total	3067	100	37.54 [37.28;37.80]	-0.70 [-0.74;-0.66]

*Note*: 95% confidence interval in brackets. Trajectories are described by 1 for exposure to low OP, and 0 for no exposure. Averaged across countries and predicted for the level of the cohorts 1931–1933.

**Table 5:** Predictions of intercept and slope of grip strength by life course OP pattern (kg)–Women.

Trajectory	N	(%)	Intercept	Slope
000	1354	66.34	23.75 [22.83;24.67]	-0.42 [-0.57;-0.28]
001	69	3.38	23.24 [21.76;24.72]	-0.31 [-0.53;-0.09]
010	74	3.63	24.79 [23.29;26.29]	-0.44 [-0.67;-0.21]
011	196	9.60	23.71 [22.53;24.89]	-0.44 [-0.62;-0.26]
100	149	7.30	23.74 [22.56;24.92]	-0.42 [-0.60;-0.23]
101	13	0.64	25.61 [22.55;28.67]	-0.71 [-1.11;-0.31]
110	38	1.86	25.12 [23.30;26.95]	-0.38 [-0.65;-0.11]
111	148	7.25	23.33 [22.06;24.60]	-0.37 [-0.57;-0.17]
Total	2041	100	23.60 [23.38;23.82]	-0.42 [-0.45;-0.39]

*Note*: 95% confidence interval in brackets. Trajectories are described by 1 for exposure to low OP, and 0 for no exposure. Averaged across countries and predicted for the level of the cohorts 1931–1933.

In the lower part of Fig 2 and in Table 5 we can see that both slopes and levels of grip strength are much closer together for women than for men. This indicates only small differences related to life course OP. The only trajectories that have notably higher levels of grip strength are (110) and (010), containing those who are intra-generationally upwardly mobile and those who are exposed only in young adulthood, respectively. Note, however, that these categories contain only few women.

In the third step of our analysis, we conducted Wald tests on the restrictions of the coefficients that reflect the different life-course models. The technical description of these restrictions can be found in Table A of S1 Appendix. For the level of grip strength for men, the best model is a critical period in midlife showing the highest p-value and lowest AIC (Tables 6 and 7). Based on this model, those who are in a low OP during midlife are predicted to have 1.67 kg (95% CI -2.33;-1.00) less grip strength in old age. Table 6 shows further that there is no significant association between life course patterns of OP and the slope of grip strength for men in the model, as the null model cannot be rejected. That means that decline in health does not differ systematically across trajectories of OP. There is no increase or decrease in differences; health inequalities remain constant.

**Table 6:** Relative model fit of life course model (p-value).

Life course model	Men		Women	
	Intercept	Slope	Intercept	Slope
Null	0.00	0.09	0.26	0.58
Accumulation	0.03	0.06	0.18	0.49
Social mobility—early	0.01	0.03	0.15	0.40
Social mobility—late	0.00	0.11	0.68	0.39
Social mobility–any	0.06	0.08	0.32	0.36
Critical period—childhood	0.00	0.07	0.18	0.49
Critical period–young adulthood	0.11	0.06	0.20	0.47
Critical period—midlife	0.66	0.08	0.24	0.49
Sensitive period	0.87	0.09	0.29	0.27

*Note*: p-values are calculated based on Wald tests on parameter constraints. The null model needs to show a value below 0.05. Higher value indicates better model fit relative to the saturated model (for technical details see S1 Appendix). The technical description of the restrictions for the models can be found in Table A of S1 Appendix.

**Table 7:** Relative model fit of life course model (AIC).

Life course model	Men		Women	
	Intercept	Slope	Intercept	Slope
Null	81884	81882	50474	50470
Accumulation	81896	81880	50472	50469
Social mobility—early	81887	81884	50475	50472
Social mobility—late	81898	81881	50470	50472
Social mobility–any	81883	81882	50473	50473
Critical period—childhood	81898	81882	50474	50470
Critical period–young adulthood	81880	81882	50474	50471
Critical period—midlife	**81874**	81881	50473	50470
Sensitive period	81875	81882	50474	50474

*Note*: p-values are calculated based on Wald tests on parameter constraints. The null model needs to show a value below 0.05. Higher value indicates better model fit relative to the saturated model (for technical details see S1 Appendix). Best fit given rejection of the null model is marked as bold. The technical description of the restrictions for the models can be found in Table A of S1 Appendix.

For women, the application of the life course model tests confirms the impression given by Fig 2. Both the levels of and decline in physical health are not related systematically to women’s OP trajectory (Table 6).

To check the sensitivity of our results to the coding of the exposure to low OP variable, we reran the analyses, with exposure defined as a blue-collar occupation (ISCO major groups 6–9) versus all white-collar occupations yielding very similar results (Tables D-G in S1 Appendix). In addition, we also reran our models to include those individuals with missing information for 1 or 2 periods. The results remain stable (Tables L–O in S1 Appendix). As additional sensitivity checks, we controlled separately for height and weight, which also did not change our results (Tables H-K in S1 Appendix).

## Discussion

In this study we investigated the influence of low OP over the life course on levels and decline in grip strength at old age. For men, the results of the generalized structured regression approach suggest that exposure to low OP is especially harmful during midlife. For women, no relevant association to life course OP could be found.

For men, the results stand in contrast to other studies which stress the importance of early life exposure to low OP and different dimensions of health[15,47–51], because taking the different trajectories into account did not reveal a relevant influence of parental OP during childhood on grip strength in old age. However, it should be noted that there are also other studies which do not find an association of childhood OP and grip strength[52].

We found that the difference in physical function between those exposed during peak working age and those unexposed was already established at the age of 65, and our analysis provided no evidence for any of the life course theories suggesting convergence (age-as-leveler) or divergence (cumulative disadvantage) after that age. One possible explanation might be that exposure to low OP reduces maximum attainable strength (functional reserve)[53], but does not affect the rate of decline in old age. An argument against this proposition is that studies have shown that maximum grip strength is usually reached before the age of 35[26], which is before the critical period of exposure. Consequently, it seems more likely that there is differential decline in grip strength during the period of later working life, favoring those who are not exposed. To the extent that our indicator of OP capture more directly working life experience our results then could actually be seen as being in line with the cumulative disadvantage since those earlier exposed are now retired from the labour market and therefore not directly exposed. More importantly is to stress that our findings with similar decline from different levels of grip strength imply that inequalities in physical function continues also at advanced old age. Both in research and practice, it is consequently important to highlight the heterogeneity in old-age health and the impact of social stratification.

Previous studies for adult and old-age health have found mixed support for the gender difference in influence of OP on physical function; some find a similar association for men and women[39,54], some find weaker or no association for women[38], as in our study. The clear gender difference found in our study stresses the importance of gender-specific analysis, because both trajectories and their health consequences might be different for men and women[55].

One explanation for the lack of association between life course OP and grip strength in old age for women is that the women in the cohorts under investigation who are continuously active on the labor market represent a health-selected population. When the male breadwinner model is dominant, women with health limitations are more likely to drop out of employment. This might suppress a possible association between their life course OP and health in old age. Accordingly, a study adopting the household as the unit to define OP, what is normally called the dominance approach, might have led to a different finding for women [56,57].

### Strengths and limitations

In our study we combined a dynamic view on OP over the life course with a dynamic view on inequalities in physical function in old age. This gives a more comprehensive perspective on differences in aging by jointly addressing life course models of accumulation, critical period, and social mobility with theories on the development of health inequalities such as age-as-leveler and cumulative advantage. We demonstrated that the structured regression approach can be generalized to a SEM framework, allowing more flexible tests of life course models. We made use of a large representative data set that collected grip strength as an objective indicator of grip strength over five points in time. This allowed us to combine life course information on OP with estimated of the trajectories in grip strength which is rarer due to the restricted number of data sets containing information on both aspects. We took differences in likelihood of drop-out due to poor health (and death) and low grip strength into account by modeling separately for five patterns of missing values. Our study demonstrates the need and the potential to integrate different strands of theory on socially stratified processes of aging with the appropriate methods developed in different fields of longitudinal and life course research.

A recent study proposed an alternative strategy of establishing the explanatory power of life course models[58] for which an integration with LGM might be useful in future research.

Despite these strengths there are several limitations to our study. Occupational position reflects only one dimension of socioeconomic position (SEP) of individuals. Usually, income, education, and wealth are treated as other important dimensions of SEP. It has been shown that these different dimension can have different impact on health and health inequalities in the life course [59,60] and on the trajectories of different health indicators, including functional limitations [61]. Therefore, our results should not be generalized to all aspects of SEP. Instead further research could look at trajectories in other dimensions. The limitations are that education is usually time-constant at a certain age, early in the life of individuals. Wealth on the other hand is by definition the outcome of a cumulative process, increasing for most individuals throughout the life course. Classical upward or downward mobility patterns therefore do not apply to this dimension. Last, income could be analyzed in a similar fashion, but here data availability is the problem as information about income over the whole life course (including parental income) is still hard to acquire in combination with old age health outcomes. Improvements in survey data and cohort studies might allow replications of our analyses with trajectories of income as the determinant of physical functioning in old age.

The dichotomization is on the one hand necessary to ensure that the trajectories do not become overly complex. On the other hand using a dichotomous indicator will hide a lot of variation within the categories. However, we showed that a different coding into blue-collar and white-collar workers did not change the results. Comparative work with alternative indicators of OP should be added in the future. It should be further noted that by definition the use of an occupational indicator limits the analyses to the employed population. Alternative indicators of SEP like trajectories of household income could be used in future studies to get estimates for the non-employed as well.

The use of retrospective data has several advantages and disadvantages for our study. Despite the fact that SHARE uses a life grid approach that is designed to maximize accuracy in remembering occurrence and temporal ordering of events in the life course[24,62], retrospective data faces the problem of incorrect recall of occupational status[63–65]. In contrast to many prospective cohort studies, we have continuous yearly information on occupational status from age 16 onwards. That allows us to average occupational status during adulthood and midlife respectively, which should reduce measurement error and recall bias. Another advantage of retrospective data is that there are no drop-outs during the observation of OP in the life course[66–68]. We face the problem of increasing selectivity only to a small degree, as there are only 2 waves in which drop-out can occur before OP is measured for the whole life course. Still, it needs to be acknowledged that we might have a positively-selected sample with regard to (decline in) health if those who dropped out before answering the retrospective questionnaire in wave 3 have lower health overall, or a stronger health decline. This might lead to an underestimation of the association between life course occupational position with grip strength in our study. Additionally, our sample only includes survivors to old age. This means that mortality in early and midlife can lead to a reduction in health inequalities that we do not observe. Thus, the results only apply to those who survived until the age of 65 and should not be generalized to younger ages. A further problem might arise if childhood OP has a higher degree of error than midlife OP. In this case the relative strength of the association of childhood OP with grip strength might be underestimated compared to the association of midlife OP with grip strength.

One possible concern with using grip strength related to an occupational indicator is that workers in elementary occupations have physically more demanding jobs, which could lead to a training effect of their muscles. After retirement they could therefore have a higher decline as they no longer engage in physical labor, which would reflect detraining, and not decline in grip strength. We do not believe that this constitutes a source of bias. Firstly, the literature on detraining shows that detraining happens very quickly, i.e. training effects vanish almost completely after half a year, often earlier, depending on the training treatment[69–71]. The sample consists of those who are no longer working, and therefore any possible detraining will, for the most part, have happened before the observation begins. Second, there is little evidence that suggests that blue-collar workers or workers who have a high physical workload have higher grip strength than white-collar workers[26,72–75], a result that we can replicate with our data for those individuals below 65 who are still active on the labor market. Finally, it is important to note that the results cannot be interpreted as causal estimates in the counter-factual sense. It is possible that there are common factors for both trajectories in occupational position and level and development of grip strength in old age (e.g. physiological dispositions acquired very early in life). The results should be regarded as associations representing the total effect of life course OP on grip strength, including direct and mediated associations, and possible selection effects during the life course.

## Conclusions

Combining a dynamic perspective on both life course OP and grip strength in old age provides a good view on the pattern of health inequalities in old age. For men, mid-life exposure to low OP correlates with decline in grip strength. No further convergence or divergence could be found during old age. As grip strength is a reliable indicator of other aspects of physical functioning we would expect to see similar results in the association of life course OP and other indicators of physical functioning. Furthermore, it is important to look at socioeconomic trajectories of men and women separately. Our extension of the structured regression approach to SEM and LGM can be used in future research in life course epidemiology.

## Supporting Information

S1 Appendix Technical details on models and results of sensitivity analyses. (PDF)

S1 Code Code to replicate all analyses conducted in the study. (ZIP)
