# Supplementary material for: The Association of Levels of and Decline in Grip Strength in Old Age with Trajectories of Life Course Occupational Position
Source: PLoS One. 2016 May 27;11(5):e0155954. doi: 10.1371/journal.pone.0155954 (PMC4883757; doi:10.1371/journal.pone.0155954)
Supplement: S1 Appendix — (PDF) [file pone.0155954.s001.pdf]

The association of level and decline in grip strength in old age with trajectories of life course occupational position

Hannes Kröger, Johan Fritzell, Rasmus Hoffmann

S1 – Appendix

<u>**Table of Contents**</u>

Formulas for the estimation of the LGM and the generalization of SRA2
Results from the latent growth model 4
Results for blue collar workers versus white collars workers 6
Results controlling for height and weight 10
Results including individuals with one or two missing values on OP 14
Results using missing at random assumption (MAR) 18

Formulas for the estimation of the LGM and the generalization of SRA

The LGM is defined in the following way:

$$\boldsymbol{y}_i = \boldsymbol{\Lambda}\boldsymbol{\eta}_i + \boldsymbol{\epsilon}_i$$

$\boldsymbol{\eta}_i = \boldsymbol{\mu}_\eta + \boldsymbol{\zeta}_i$ (1)

The index i refers to the observed individuals. $\boldsymbol{y}$ is the vector of the five observed measures of grip strength, $\boldsymbol{\Lambda}$ is the vector of constraints, identifying intercept and slope. $\boldsymbol{\epsilon}_i$ represents the vector of individual specific errors in grip strength. $\boldsymbol{\eta}_i$ contains the values of each individual on the intercept and slope parameter, $\boldsymbol{\mu}_\eta$ holds the means of the intercept and slope, representing level and decline in physical functioning, and $\boldsymbol{\zeta_i}$ is the individual deviation from the mean of the intercept and slope, representing the variability in level and decline of physical functioning.
Instead of using the waves as fixed time points we use month specific age at each wave as an individually varying-time point. This increases the precision of estimates and allows us to make age related statements instead of period related statements. This means that Λ takes the form:

$$\Lambda = \begin{bmatrix} 1 & age_{1i} \\ 1 & age_{2i} \\ 1 & age_{3i} \\ 1 & age_{4i} \\ 1 & age_{5i} \end{bmatrix}$$

Age is centered at 75, so that all reports of the intercept refer to this age. Applying the pattern-mixture approach to account for non-random drop-out the mean of the intercept and slope of the LGM are estimated as the weighted average of the intercept and slope of each of the five missing patterns, where π_p is the proportion of pattern p of the total sample:

In our structural equation model the intercept and slope are predicted by exposure in the three periods of life course and all their interactions ($\boldsymbol{X_i}$) and controlled for differences between countries and cohorts ($\boldsymbol{C}_i$) :

$\boldsymbol{\eta}_i = \boldsymbol{\mu}_\eta + \boldsymbol{X_i}\boldsymbol{\beta}_\eta + \boldsymbol{C}_i\boldsymbol{\gamma}_\eta + \boldsymbol{\zeta}_i$ (2)

The vector of coefficients is independently estimated for intercept and slope. Applying the pattern-mixture approach to account for non-random drop-out the mean of the intercept and slope of the LGM and their association with countries and life course SES are estimated as the weighted average of the respective estimates in each of the five missing patterns. Note that for identification purposes two parameters reflecting unique effect of life course trajectory (101) and (010) had to be constrained to be equal across missing patterns. π_p is the proportion of pattern p of the total sample:

$$\boldsymbol{\mu}_\eta = \pi_1 * \boldsymbol{\mu}_{\eta 1} + \pi_2 * \boldsymbol{\mu}_{\eta 2} + \pi_3 * \boldsymbol{\mu}_{\eta 3} + \pi_4 * \boldsymbol{\mu}_{\eta 4} + \pi_5 * \boldsymbol{\mu}_{\eta 5}$$
$$\boldsymbol{\beta}_\eta = \pi_1 * \boldsymbol{\beta}_{\eta 1} + \pi_2 * \boldsymbol{\beta}_{\eta 2} + \pi_3 * \boldsymbol{\beta}_{\eta 3} + \pi_4 * \boldsymbol{\beta}_{\eta 4} + \pi_5 * \boldsymbol{\beta}_{\eta 5}$$

$$\boldsymbol{\gamma}_\eta = \pi_1 * \boldsymbol{\gamma}_{\eta 1} + \pi_2 * \boldsymbol{\gamma}_{\eta 2} + \pi_3 * \boldsymbol{\gamma}_{\eta 3} + \pi_4 * \boldsymbol{\gamma}_{\eta 4} + \pi_5 * \boldsymbol{\gamma}_{\eta 5}$$

To test for life course patterns, equation (2) is restricted as described in Mishra et al. [1] for accumulation, critical period and social mobility and as described in Mishra et al. [2] for sensitive periods (see below for details). For each of the set of restrictions representing one life course model M, the Wald statistic is then calculated as:

$$\boldsymbol{W}_{\eta M} = \boldsymbol{R_M \beta'}_\eta \times \left(\boldsymbol{R}_M \boldsymbol{V}_{\beta_\eta} \boldsymbol{R'}_M\right)^{-1} \boldsymbol{R\beta}_\eta, \boldsymbol{W}_{\eta M} \sim \chi^2_{q_M} \text{ (3)}$$

$\boldsymbol{V}_{\beta_\eta}$ is the variance-covariance matrix of the estimated coefficients $\boldsymbol{\beta}_\eta$ and q_M the degrees of freedom depending on the life course model. Based on the Wald-statistic the p-values are calculated from χ^2 distribution with the respective degrees of freedom. The Akaike information criterion (AIC) is calculated as:

$$\boldsymbol{AIC_{\eta M}} = AIC_{sat} + \boldsymbol{W}_{\eta M} - 2q_M$$

Table A. The technical restrictions that are used are the following:

**Life course model**	**Coefficient restriction**
Null-model	$\beta_1 = \beta_2 = \beta_3 = \beta_{12} = \beta_{13} = \beta_{23} = \beta_{123} = 0$
Accumulation	$\beta_1 = \beta_2 = \beta_3; \beta_{12} = \beta_{13} = \beta_{23} = \beta_{123} = 0$
Critical period (childhood)	$\beta_2 = \beta_3 = \beta_{12} = \beta_{13} = \beta_{23} = \beta_{123} = 0$
Critical period (early adulthood)	$\beta_1 = \beta_3 = \beta_{12} = \beta_{13} = \beta_{23} = \beta_{123} = 0$
Critical period (midlife)	$\beta_1 = \beta_2 = \beta_{12} = \beta_{13} = \beta_{23} = \beta_{123} = 0$
Social mobility (inter-generational)	$\beta_{23} = -(\beta_2 + \beta_3); \beta_1 = \beta_{12} = \beta_{13} = \beta_{123} = 0$
Social mobility (intra-generational)	$\beta_{12} = -(\beta_1 + \beta_2); \beta_3 = \beta_{13} = \beta_{23} = \beta_{123} = 0$
Social mobility (any)	$\beta_2 = (\beta_1 + \beta_3); \beta_{12} = \beta_{23} = -\beta_2; \beta_{13} = \beta_{123} = 0$
Sensitive Period	$\beta_{12} = \beta_{13} = \beta_{23} = \beta_{123} = 0$

1. Mishra G, Nitsch D, Black S, Stavola BD, Kuh D, Hardy R. A structured approach to modelling the effects of binary exposure variables over the life course. *Int J Epidemiol*. 2009 Jan 4;**38**(2):528–537.
2. Mishra GD, Chiesa F, Goodman A, Stavola BD, Koupil I. Socio-economic position over the life course and all-cause, and circulatory diseases mortality at age 50–87 years: results from a Swedish birth cohort. *Eur J Epidemiol*. 2013;**28**(2):139–147.

Results from the latent growth model

Table B. Mean and covariance structure of the latent growth model with linear slope

Parameter	Women	Men
Mean(Intercept)	23.60	37.54
	[23.38;23.82]	[37.28;37.80]
Mean(Slope)	-0.42	-0.70
	[-0.45;-0.39]	[-0.74;-0.66]
Variance(Intercept)	19.36	40.42
	[17.73;20.98]	[37.63;43.21]
Variance(Slope)	0.03	0.09
	[0.00;0.06]	[0.04;0.14]
Correlation(Intercept.Slope)	-0.11	0.04
	[-0.28;0.07]	[-0.09;0.17]
e.Variance(grip strength wave 1)	13.40	20.64
	[11.30;15.49]	[17.97;23.30]
e.Variance(grip strength wave 2)	19.19	28.09
	[15.91;22.47]	[24.60;31.58]
e.Variance(grip strength wave 3)	20.12	34.13
	[16.39;23.85]	[28.43;39.82]
e.Variance(grip strength wave 4)	8.74	19.94
	[7.53;9.94]	[16.99;22.90]
e.Variance(grip strength wave 5)	10.57	17.66
	[8.07;13.07]	[14.99;20.34]

Note: Means are reported in kg. The mean of the intercept refers to the average level of grip strength at age 75. The mean of the slope refers to the average decline per year in grip strength.

Table C. Model fit (BIC) of three specifications of the slope for the latent growth model of grip strength using missing at random specification instead of a pattern-mixture model

Slope	Linear	Quadratic	Linear semi-parametric
Men	72813	**72804**	72829
Women	44081	**44080**	44090

Note: Bold numbers indicate best model fit. While quadratic fit is slightly better than linear, it does not justify the additional complexity of the model.

Results for blue collar workers versus white collars workers

Table D. Predictions of intercept and slope of grip strength by life course OP pattern (kg) - Men

Trajectory	N	(%)	Intercept (at age 75)	Slope (decline per year)
000	517	16.86	38.43	-0.73
			[37.33;39.53]	[-0.89;-0.56]
001	17	0.55	34.59	-0.75
			[31.18;38.00]	[-1.23;-0.28]
010	35	1.14	39.06	-0.77
			[37.04;41.08]	[-1.05;-0.49]
011	120	3.91	38.99	-0.76
			[37.46;40.52]	[-0.98;-0.54]
100	659	21.49	38.54	-0.70
			[37.46;39.63]	[-0.87;-0.54]
101	47	1.53	37.98	-0.47
			[36.10;39.86]	[-0.76;-0.17]
110	207	6.75	39.01	-0.73
			[37.64;40.38]	[-0.94;-0.52]
111	1465	47.77	37.56	-0.72
			[36.53;38.59]	[-0.88;-0.56]
Total	3067	100	37.54	-0.70
			[37.28;37.80]	[-0.66;-0.74]

Note: 95% confidence interval in brackets. Trajectories are described by 1 for exposure to low OP, and 0 for no exposure. Averaged across countries and predicted for the level of the cohorts 1931-1993. OP is coded as blue-collar (ISCO major groups 6-9) versus white collar workers.

Table E. Predictions of intercept and slope of grip strength by life course OP pattern (kg) - Women

Trajectory	N	(%)	Intercept (at age 75)	Slope (decline per year)
000	394	19.30	23.93	-0.43
			[22.88;24.97]	[-0.59;-0.28]
001	22	1.08	24.42	-0.45
			[22.19;26.65]	[-0.73;-0.16]
010	21	1.03	21.50	-0.42
			[19.13;23.87]	[-0.78;-0.06]
011	61	2.99	23.66	-0.37
			[22.07;25.24]	[-0.57;-0.17]
100	658	32.24	23.83	-0.39
			[22.82;24.83]	[-0.54;-0.24]
101	52	2.55	23.60	-0.42
			[21.83;25.36]	[-0.65;-0.19]
110	127	6.22	24.40	-0.44
			[23.07;25.73]	[-0.64;-0.23]
111	706	34.59	23.57	-0.42
			[22.49;24.64]	[-0.58;-0.27]
Total	2041	100	23.60	-0.42
			[23.38;23.82]	[-0.45;-0.39]

Note: 95% confidence interval in brackets. Trajectories are described by 1 for exposure to low OP, and 0 for no exposure. Averaged across countries and predicted for the level of the cohorts 1931-1993. OP is coded as blue-collar (ISCO major groups 6-9) versus white collar workers.

Table F. Relative model fit of life course model (p-value)

Life course model	Men		Women	
	Intercept	Slope	Intercept	Slope
Null	0.00	0.74	0.35	0.98
Accumlation	0.03	0.63	0.32	0.96
Social mobility - early	0.01	0.68	0.29	1.00
Social mobility - late	0.00	0.90	0.18	0.93
Social mobility – any	0.01	0.57	0.23	0.97
Critical period - Childhood	0.00	0.68	0.26	0.96
Critical period – young adulthood	0.00	0.69	0.32	0.96
Critical period - midlife	0.13	0.63	0.35	0.96
Sensitive Periods	0.10	0.68	0.16	0.87

Note: p-values are calculated based on Wald-tests on parameter constraints. The null model needs to show a value below 0.05. Higher value indicates better model fit relative to the saturated model (for technical details see appendix). SEP is coded as blue-collar (ISCO major groups 6-9) versus white collar workers.

Table G. Relative model fit of life course model (AIC)

Life course model	Men		Women	
	Intercept	Slope	Intercept	Slope
Null	81907	81887	50489	50483
Accumlation	81899	81889	50490	50485
Social mobility - early	81903	81890	50492	50486
Social mobility - late	81905	81889	50493	50487
Social mobility – any	81902	81891	50492	50486
Critical period - Childhood	81906	81889	50491	50485
Critical period – young adulthood	81904	81889	50490	50485
Critical period - midlife	**81895**	81889	50490	50485
Sensitive Period	81897	81891	50494	50489

Note: p-values are calculated based on Wald-tests on parameter constraints. The null model needs to show a value below 0.05. Higher value indicates better model fit relative to the saturated model (for technical details see appendix). Best fit given rejection of the Null model is marked as bold. SEP is coded as blue-collar (ISCO major groups 6-9) versus white collar workers.

Results controlling for height and weight

Table H. Predictions of intercept and slope of grip strength by life course OP pattern (kg) - Men

Trajectory	N	(%)	Intercept (at age 75)	Slope (decline per year)
000	2125	69.29	38.06	-0.69
			[37.10; 39.02]	[-0.84; -0.54]
001	62	2.02	37.04	-0.63
			[34.92; 39.17]	[-0.88; -0.38]
010	83	2.71	37.82	-0.89
			[36.31; 39.34]	[-1.12; -0.66]
011	208	6.78	36.91	-0.57
			[35.61; 38.20]	[-0.76; -0.37]
100	315	10.27	38.63	-0.73
			[37.44; 39.83]	[-0.92; -0.53]
101	17	0.55	37.13	-0.44
			[33.01; 41.25]	[-0.89; 0.00]
110	56	1.83	38.55	-0.60
			[36.38; 40.72]	[-0.93; -0.27]
111	201	6.55	36.67	-0.76
			[35.27; 38.07]	[-0.97; -0.55]
Total	3067	100	37.54	-0.70
			[37.28;37.80]	[-0.66;-0.74]

Note: 95% confidence interval in brackets. Trajectories are described by 1 for exposure to low OP, and 0 for no exposure. Averaged across countries and predicted for the level of the cohorts 1931-1993. OP is coded as elementary occupations (ISCO major group 9) versus white collar workers. Controlled for height and weight.

Table I. Predictions of intercept and slope of grip strength by life course OP pattern (kg) - Women

Trajectory	N	(%)	Intercept (at age 75)	Slope (decline per year)
000	1354	66.34	23.69	-0.43
			[22.79; 24.60]	[-0.57; -0.29]
001	69	3.38	23.23	-0.30
			[21.78; 24.67]	[-0.52; -0.09]
010	74	3.63	24.39	-0.43
			[23.09; 25.68]	[-0.64; -0.22]
011	196	9.60	23.80	-0.45
			[22.67; 24.93]	[-0.61; -0.29]
100	149	7.30	23.81	-0.45
			[22.68; 24.94]	[-0.63; -0.27]
101	13	0.64	25.19	-0.69
			[22.82; 27.56]	[-1.04; -0.33]
110	38	1.86	24.91	-0.41
			[23.01; 26.81]	[-0.71; -0.12]
111	148	7.25	23.33	-0.38
			[21.99; 24.67]	[-0.58; -0.19]
Total	2041	100	23.60	-0.42
			[23.38;23.82]	[-0.45;-0.39]

Note: 95% confidence interval in brackets. Trajectories are described by 1 for exposure to low OP, and 0 for no exposure. Averaged across countries and predicted for the level of the cohorts 1931-1993. OP is coded as elementary occupations (ISCO major group 9) versus white collar workers. Controlled for height and weight.

Table J. Relative model fit of life course model (p-value)

Life course model	Men		Women	
	Intercept	Slope	Intercept	Slope
Null	0.01	0.10	0.44	0.49
Accumlation	0.14	0.06	0.34	0.38
Social mobility - early	0.06	0.04	0.35	0.36
Social mobility - late	0.00	0.12	0.64	0.34
Social mobility – any	0.18	0.12	0.57	0.30
Critical period - Childhood	0.01	0.08	0.34	0.37
Critical period – young adulthood	0.21	0.06	0.39	0.37
Critical period - midlife	0.84	0.10	0.35	0.39
Sensitive Periods	0.85	0.12	0.33	0.19

Note: p-values are calculated based on Wald-tests on parameter constraints. The null model needs to show a value below 0.05. Higher value indicates better model fit relative to the saturated model (for technical details see appendix). OP is coded as elementary occupations (ISCO major group 9) versus white collar workers. Controlled for height and weight.

Table K. Relative model fit of life course model (AIC)

Life course model	Men		Women	
	Intercept	Slope	Intercept	Slope
Null	81117	81111	49729	49729
Accumlation	81111	81113	49731	49731
Social mobility - early	81114	81115	49732	49732
Social mobility - late	81120	81112	49730	49732
Social mobility – any	81111	81112	49730	49733
Critical period - Childhood	81119	81113	49731	49731
Critical period – young adulthood	81110	81113	49731	49731
Critical period - midlife	**81104**	81112	49731	49731
Sensitive Period	81106	81112	49733	49735

Note: p-values are calculated based on Wald-tests on parameter constraints. The null model needs to show a value below 0.05. Higher value indicates better model fit relative to the saturated model (for technical details see appendix). Best fit given rejection of the Null model is marked as bold. OP is coded as elementary occupations (ISCO major group 9) versus white collar workers. Controlled for height and weight.

Results including individuals with one or two missing values on OP

Table L. Predictions of intercept and slope of grip strength by life course OP pattern (kg) - Men

Trajectory	N	(%)	Intercept (at age 75)	Slope (decline per year)
000	517		38.10	-0.69
			[37.19; 39.01]	[-0.83; -0.56]
001	17		36.88	-0.65
			[35.20; 38.57]	[-0.82; -0.48]
010	35		37.47	-0.90
			[35.84; 39.10]	[-1.14; -0.66]
011	120		36.77	-0.59
			[35.46; 38.08]	[-0.78; -0.40]
100	659		38.88	-0.73
			[37.71; 40.04]	[-0.90; -0.55]
101	47		37.48	-0.40
			[34.49; 40.46]	[-0.82; 0.01]
110	207		38.39	-0.58
			[36.43; 40.36]	[-0.87; -0.28]
111	1465		36.60	-0.78
			[35.28; 37.92]	[-0.97; -0.58]
Total	3697	100	37.20	-0.72
			[36.96; 37.45]	[-0.68;-0.75]

Note: 95% confidence interval in brackets. Trajectories are described by 1 for exposure to low OP, and 0 for no exposure. Averaged across countries and predicted for the level of the cohorts 1931-1993. OP is coded as elementary occupations (ISCO major group 9) versus white collar workers. The number of observations of the trajectories does not add up to the total number of observations, because the individuals included who have one or two missing values on OP do not have a fully observed trajectory. However, they contribute to the individual coefficient estimation.

Table M. Predictions of intercept and slope of grip strength by life course OP pattern (kg) - Women

Trajectory	N	(%)	Intercept (at age 75)	Slope (decline per year)
000	2125		23.73	-0.42
			[22.90; 24.55]	[-0.56; -0.28]
001	62		23.52	-0.31
			[22.27; 24.77]	[-0.50; -0.12]
010	83		24.89	-0.43
			[23.46; 26.32]	[-0.66; -0.21]
011	208		23.63	-0.41
			[22.53; 24.73]	[-0.58; -0.24]
100	315		23.81	-0.41
			[22.71; 24.92]	[-0.59; -0.22]
101	17		25.41	-0.64
			[22.41; 28.42]	[-1.03; -0.24]
110	56		25.32	-0.38
			[23.56; 27.08]	[-0.64; -0.11]
111	201		23.32	-0.34
			[22.14; 24.49]	[-0.53; -0.15]
Total	3374	100	23.34	-0.42
			[23.12;23.48]	[-0.40;-0.45]

Note: 95% confidence interval in brackets. Trajectories are described by 1 for exposure to low OP, and 0 for no exposure. Averaged across countries and predicted for the level of the cohorts 1931-1993. OP is coded as elementary occupations (ISCO major group 9) versus white collar workers. The number of observations of the trajectories does not add up to the total number of observations, because the individuals included who have one or two missing values on OP do not have a fully observed trajectory. However, they contribute to the individual coefficient estimation.

Table N. Relative model fit of life course model (p-value)

Life course model	Men		Women	
	Intercept	Slope	Intercept	Slope
Null	0.00	0.05	0.18	0.60
Accumlation	0.03	0.03	0.12	0.60
Social mobility - early	0.03	0.02	0.09	0.38
Social mobility - late	0.00	0.07	0.76	0.48
Social mobility – any	0.10	0.06	0.35	0.39
Critical period - Childhood	0.00	0.03	0.12	0.53
Critical period – young adulthood	0.10	0.03	0.13	0.51
Critical period - midlife	0.54	0.07	0.17	0.65
Sensitive Periods	0.78	0.05	0.18	0.42

Note: p-values are calculated based on Wald-tests on parameter constraints. The null model needs to show a value below 0.05. Higher value indicates better model fit relative to the saturated model (for technical details see appendix). OP is coded as elementary occupations (ISCO major group 9) versus white collar workers. Analyses include those individuals who have one or two missing values on OP.

Table O. Relative model fit of life course model (AIC)

Life course model	Men		Women	
	Intercept	Slope	Intercept	Slope
Null	76157	76147	49340	49336
Accumlation	76148	76148	49342	49337
Social mobility - early	76149	76150	49344	49340
Social mobility - late	76159	76147	49337	49339
Social mobility – any	76146	76147	49340	49339
Critical period - Childhood	76159	76148	49342	49337
Critical period – young adulthood	76145	76149	49342	49337
Critical period - midlife	**76139**	76146	49341	49336
Sensitive Period	76140	76148	49343	49340

Note: p-values are calculated based on Wald-tests on parameter constraints. The null model needs to show a value below 0.05. Higher value indicates better model fit relative to the saturated model (for technical details see appendix). Best fit given rejection of the Null model is marked as bold. OP is coded as elementary occupations (ISCO major group 9) versus white collar workers. Analyses include those individuals who have one or two missing values on OP.

Results using missing at random assumption (MAR)

Table P. Relative model fit of life course model (p-value)

Life course model	Men		Women	
	Intercept	Slope	Intercept	Slope
Null	0.00	0.35	0.20	0.57
Accumlation	0.02	0.27	0.14	0.47
Social mobility - early	0.00	0.18	0.11	0.41
Social mobility - late	0.00	0.22	0.23	0.39
Social mobility – any	0.02	0.22	0.24	0.33
Critical period - Childhood	0.00	0.30	0.15	0.45
Critical period – young adulthood	0.06	0.27	0.14	0.51
Critical period - midlife	0.45	0.26	0.21	0.45
Sensitive Periods	0.44	0.17	0.14	0.29

Note: p-values are calculated based on Wald-tests on parameter constraints. The null model needs to show a value below 0.05. Higher value indicates better model fit relative to the saturated model (for technical details see appendix). OP is coded as elementary occupation (ISCO major groups 9) versus all other occupations.

Table Q. Relative model fit of life course model (AIC)

Life course model	Men		Women	
	Intercept	Slope	Intercept	Slope
Null	72345	72325	43932	43928
Accumlation	72333	72326	43934	43930
Social mobility - early	72339	72328	43935	43931
Social mobility - late	72346	72328	43933	43931
Social mobility – any	72335	72328	43933	43932
Critical period - Childhood	72347	72326	43934	43930
Critical period – young adulthood	72331	72326	43934	43929
Critical period - midlife	**72325**	72327	43933	43930
Sensitive Period	72327	72329	43935	43933

Note: p-values are calculated based on Wald-tests on parameter constraints. The null model needs to show a value below 0.05. Higher value indicates better model fit relative to the saturated model (for technical details see appendix). Best fit given rejection of the Null model is marked as bold. OP is coded as elementary occupation (ISCO major group 9) versus all others.
